# Relationship between meaning in life and death anxiety in the elderly: self-esteem as a mediator

**DOI:** 10.1186/s12877-019-1316-7

**Published:** 2019-11-12

**Authors:** Jiaxi Zhang, Jiaxi Peng, Pan Gao, He Huang, Yunfei Cao, Lulu Zheng, Danmin Miao

**Affiliations:** 1 Xi’an Research Institute of High-technology, Xi’an, China; 2 College of Teachers, Chengdu University, Chengdu, China; 3 Department of Military Medical Psychology, Air Force Military Medical University, Xi’an, China

**Keywords:** Meaning in life, Death anxiety, Self-esteem, Mental health, Older adults

## Abstract

**Background:**

Death anxiety is a common phenomenon in all societies. Older adults may be more prone to death anxiety than their younger counterparts; however, death anxiety among older adults is not well understood. This study explores the relationship between meaning in life, self-esteem, and death anxiety in senior citizens in China.

**Methods:**

A total of 283 older adults participated in this study; data were collected via the Meaning in Life Questionnaire, the Rosenberg Self-Esteem Scale, and the Death Anxiety Scale.

**Results:**

Results show that the dimensions of meaning in life, presence of meaning (*r =* − 0.43, *p <* 0.01), search for meaning (*r =* − 0.31, *p <* 0.01), and self-esteem (*r =* − 0.54, *p <* 0.01) were each negatively correlated with death anxiety. Regression analysis reveals that meaning in life significantly predicted self-esteem and death anxiety (*F =* 45.70, *p <* 0.01; R^2^ = 0.33). Path analysis indicated that self-esteem either completely or partially mediated the effects of meaning in life on death anxiety in older adults.

**Conclusions:**

Overall, meaning in life appears to be significantly correlated with death anxiety in older adults, and self-esteem can mediate this effect.

## Background

Older adults and the aging population must face the prospect of death [1]. Death is an inevitable aspect of life; as older adults approach it, they can easily experience anxiety, a reduced sense of safety, and even strong fear [2, 3]. In the psychology field, this mental state is termed *death anxiety*. Death anxiety is a conscious or unconscious psychological state resulting from a defense mechanism that can be triggered when people feel threatened by death [4]. The North American Nursing Diagnosis Association defines death anxiety as a feeling of unsafety, anxiety, or fear related to death or near-death [5]. Death anxiety is a common phenomenon in all societies, although older adults may be more prone to anxiety and fear when encountering death-related events compared to their younger counterparts [6]. Thus, relieving death anxiety in older adults may play an important role in improving their mental health and quality of life.

Several theoretical models relevant to the study of death anxiety in older adults have appeared in the literature [7–9]. Examples include self-realization theories, personal construct theory, self-concept discrepancy theory, search-for-meaning theories, theories of denial, and others. Four aspects tend to be common across these models: death is conceptualized as a radical transformation and separation, annihilation of the self, a threat to the realization of life’s basic goals and propensities, and a threat to the meaningfulness of life [10]. Such diversity in theoretical approaches has led to various perspectives on death anxiety; however, these theories are accompanied by drawbacks. One weakness relates to the substantial amount of overlap among theories [10]. Florian and Mikulincer pointed out that threats to death anxiety can be replaced by two categories depending on the chosen theory: either the meaningfulness of life or self-realization [11]. More specifically, threats to death anxiety can be classified based on two factors: external causes, such as presence and search for meaning in life; and the internalization or overall evaluation of the self. Thus, to explore more efficiently the underlying psychological mechanisms of death anxiety in older adults, external causes (meaning in life) and the internalization process (self-esteem) are included in this study. The purpose of this research is to explore the relationships among death anxiety, meaning in life, and self-esteem with a focus on the mediating effect of self-esteem between meaning in life and death anxiety in Chinese older adults.

One variable related to death anxiety is meaning in life. Frazier proposed and defined *meaning in life* as the act of humans cognizing and pursuing life goals and targets [12, 13]; that is, each person possesses unique goals or targets in life and must clearly understand what to do to achieve said targets—and make an effort to do it [14]. Steger and Frazier defined meaning in life as an individual’s ability to understand life, such as understanding oneself and the outside world and adapting to it [12]. Steger conceptualized meaning in life from multiple dimensions but ultimately considered it an individual’s understanding of life and the pursuit of his/her life goals and missions. Steger then proposed a two-dimensional model of meaning in life: presence of and search for meaning [15]. *Presence of meaning*, the cognitive dimension, is defined as the degree of devotion toward one’s goals, targets, or missions in life; this dimension focuses on the outcome. *Search for meaning*, the motivational dimension, emphasizes the process by which individuals actively identify meaning and targets in life. When individuals achieve these targets, they experience meaning in life. Many measurement scales assessing meaning in life, such as the Purpose in Life Test [16] and Sense of Coherence Scale [17], have conceptualized it as a single construct; it is therefore nearly impossible to ascertain particular aspects of life meaning that may be associated with certain outcomes. Even so, most researchers have thus far supported this two-dimensional model [18].

The relationship between meaning in life and death anxiety has been studied extensively. Routledge and Juhl asked participants to imagine the scenes of their death and discovered that only those with weaker meaning in life experienced death anxiety [19]. A stronger feeling of meaning in life has been shown to correlate with a lesser degree of death anxiety [1, 19]. Tang et al. found meaning in life to be significantly and negatively correlated with death anxiety in Chinese college students: individuals who perceived greater meaning in life could accept death and hence experienced less death anxiety [20]. Lyke indicated that meaning in life can significantly and negatively predict fear of death among young people [21]. According to meaning management theory (MMT), first proposed by Wong, human beings are meaning-seeking and meaning-making creatures with two primary motivations: to survive and to find a meaning and reason for survival [22]. MMT predicts that the pursuit of meaning in life is the best way to alleviate death anxiety. MMT also assumes that people wish to live a meaningful life and that focusing on positive growth tendencies is preferable to establishing defense mechanisms against death anxiety. Put simply, if individuals believe their existence is meaningful and that they play important roles in their community, then they may not feel threatened by inevitable death [23, 24]. As discussed above, a strong theoretical link has been established between meaning in life and death anxiety.

Another psychological construct closely related to death anxiety is self-esteem, a primary factor in death anxiety management. The literature is replete with evidence that self-esteem serves as a buffer against death anxiety. Researchers using self-report and physiological indices have found that self-esteem protects individuals from death anxiety. People with low levels of self-esteem tend to experience anxiety in response to death, whereas threats to self-esteem induce death anxiety and defense of self-esteem reduces death anxiety [25–27]. Rosenblatt and colleagues reported that, when individuals were exposed to videotaped scenes of death, viewers with high self-esteem reported less anxiety or fear [28]. Other studies have indicated that death anxiety is related to low self-esteem [2, 29]. Terror management theory (TMT), which is based on work by Becker, posits that humans possess a dual-process system that serves a protective function against human awareness of vulnerability and eventual mortality [30]. TMT further predicts that people who adhere to cultural values are better shielded than others against the potential for anxiety. This model also suggests that individuals with high self-esteem will exhibit less anxiety toward death-related scenes.

Although many studies and theoretical models have indicated that meaning in life can alleviate death anxiety, with self-esteem as an important buffer against such anxiety, the trilateral relations among death anxiety, meaning in life, and self-esteem warrant further investigation. Existing theories, such as TMT, provide indirect evidence for this mediating effect. TMT argues that people’s awareness of the inevitability of death can produce anxiety. Individuals have therefore generated numerous defense mechanisms to minimize death-related anxiety. One mechanism involves conforming to the value standards endorsed by one’s cultural worldview, which affords people a sense of meaning in life that functions as a buffer against potential anxiety related to mortality salience. People are also capable of raising their self-esteem by enhancing their sense of meaning and purpose in life, which constitutes another defense mechanism against death anxiety according to TMT [31]. In short, TMT can contextualize the effects of meaning in life on death anxiety through the mediating role of self-esteem [24]. In line with TMT, the current study aims to verify the mediating role of self-esteem in the relationship between meaning in life and death anxiety based on the following hypothesis: meaning in life can influence self-esteem and reduce death anxiety through its mediating effect on self-esteem.

## Methods

### Participants and procedures

Participants comprised a convenience sample of 294 older adults (124 men, 42.1%; 170 women, 57.9%) recruited from four large communities. Data were collected in community centers. Participants ranged from 65 to 73 years (*M*_age_ = 67.16 years; *SD* = 1.82). All participants were married and retired. Nearly all participants (271; 92.3%) reported living with their spouses, children, or other relatives; 23 (7.7%) reported living alone. All participants completed questionnaires in their respective communities. Illiterate participants, who had fewer than 3 years of education and were unable to read or write (13 older adults, 4.4%), completed the questionnaires with help from the researcher. Among the 294 questionnaires that were distributed and collected, 283 were valid. Eleven questionnaires were incomplete and excluded from analysis (questionnaires were printed two-sided, but some participants failed to complete all items on both sides). Participants received a carton of eggs as compensation. This study was approved by the Committee on Human Experimentation at the authors’ university.

### Instruments

#### Meaning in life

The 10-item Meaning in Life Questionnaire was used to assess the degree to which participants felt their life was meaningful [32]. The Meaning in Life Questionnaire consists of two subscales: search for meaning in life and presence of meaning in life. Example items for the two subscales are “I am seeking a purpose or mission for my life (search)” and “I have a good sense of what makes my life meaningful (presence).” Item responses ranged from 1 = strongly disagree to 7 = strongly agree. Research has supported the reliability and validity of this scale [33, 34]. For the purpose of this study, the Meaning in Life Questionnaire was translated into Chinese and back-translated from Chinese to English to ensure accurate translation; the translation was found to have good reliability and validity [35]. In the current study, estimated internal consistencies were α = 0.74 (search) and α = 0.81 (presence).

#### Self-esteem

The Rosenberg Self-Esteem Scale, a measure of individual self-esteem, consists of 10 self-esteem-related items such as “On the whole I am satisfied with myself” and “All in all, I am inclined to feel that I am a failure.” Each item is rated on a 7-point Likert scale, ranging from 1 = strongly disagree to 7 = strongly agree [36]. The total self-esteem score is the sum of items with reverse scoring of relevant items. The Rosenberg Self-Esteem Scale was translated into Chinese by Cheung and Lau and found to have good reliability and validity [36]. To ensure translation accuracy, the scale was back-translated from Chinese to English. The estimated internal consistency was α = 0.82 in this study.

#### Death anxiety

Templer’s unidimensional Death Anxiety Scale consists of 15 items. Six items are reverse scored, such that higher scores indicate greater death anxiety. Sample items include “I am very much afraid to die” and “I seldom think of death.” In a prior study, this scale was reported to have a test–retest reliability of 0.83 and reasonable internal consistency [37]. The Templer Death Anxiety Scale was translated into Chinese and back-translated from Chinese to English to ensure accurate translation [20]; the translated measure demonstrated good reliability and validity with an estimated internal consistency of α = 0.71 in this study.

### Data analysis

Pearson correlation coefficients and hierarchical regression analyses were used to identify relationships among meaning in life, self-esteem, and death anxiety. Baron and Kenny’s recommendations were followed in testing whether self-esteem mediated the link between meaning in life and death anxiety based on hierarchical regression analyses [38]. The following process was used: first, a simple regression analysis was performed with independent variables (presence of meaning and search for meaning) predicting the dependent variable (death anxiety) to test the significance of c; second, a simple regression analysis was conducted with independent variables (presence of meaning and search for meaning) predicting the mediating variable (self-esteem) to test the significance of a; third, a simple regression analysis was carried out with the mediating variable (self-esteem) predicting the dependent variable (death anxiety) to test the significance of b; and fourth, a multiple regression analysis was conducted with the independent variables and mediating variable predicting the dependent variable. Then, path analysis was used to test the mediation effect in Amos 17.0. Finally, the bootstrap method was adopted to test the significance of indirect and direct effects in the mediating model [39].

## Results

Table 1 lists the means, descriptive statistics, and intercorrelations of all variables. Presence of meaning was positively correlated with search for meaning (*r* = 0.50, *p* < 0.01) and self-esteem (*r* = 0.52, *p* < 0.01) and negatively correlated with death anxiety (*r* = − 0.43, *p* < 0.01). Similarly, search for meaning was associated with self-esteem (*r* = 0.36, *p* < 0.01) and death anxiety (*r* = − 0.31, *p* < 0.01). Self-esteem was negatively correlated with death anxiety (*r* = − 0.54, *p* < 0.01).

**Table 1:** Descriptive statistics and inter-correlations of all variables

	1	2	3	4	Mean	SD
1. Presence of meaning	1				25.25	6.56
2. Search for meaning	0.50^**^	1			24.22	5.62
3. Self-esteem	0.52^**^	0.36^**^	1		29.79	5.13
4. Death anxiety	−0.43^**^	− 0.31^**^	− 0.54^**^	1	12.65	5.15

Note: ^**^ *p* < 0.01

Following the steps of the mediation procedure, a series of simple regression analyses (see Table 2) indicated that presence of meaning (β = − 0.37, *p* < 0.01) and search for meaning (β = − 0.13, *p* < 0.05) were each negatively associated with death anxiety (Model 1); presence of meaning (β = 0.36, *p* < 0.01) and search for meaning (β = 0.12, *p* < 0.05) were each positively correlated with self-esteem (Model 2); and self-esteem and death anxiety were negatively correlated (β = − 0.54, *p* < 0.01; Model 3). Next, hierarchical regression analysis was conducted to test the final steps of the mediation procedure. However, when self-esteem was added to the regression analysis, the significance of the relationship between presence of meaning and death anxiety declined (β = − 0.17, *p* < 0.01) and that between search for meaning and death anxiety became insignificant (β = − 0.07, *p* = 0.22). According to Baron and Kenny [38], these results indicate partial mediation between presence of meaning and death anxiety and complete mediation between search for meaning and death anxiety (Table 2).

**Table 2:** Multiple regression analysis

Model	Dependent	Predictors	Model summary		Coefficients		
			*F*	*R*^*2*^	B	SE	*β*
1	Death anxiety	Presence of meaning	34.54^**^	0.20	−0.29	0.05	−0.37^**^
		Search for meaning			−0.11	0.06	−0.13^**^
2	Self-esteem	Presence of meaning	54.87^**^	0.28	0.36	0.04	0.46^**^
		Search for meaning			0.12	0.05	0.13^**^
3	Death anxiety	Self-esteem	117.73^**^	0.30	−0.55	0.05	−0.54^**^
4	Death anxiety	Presence of meaning	45.70^**^	0.33	−0.14	0.05	−0.17^**^
		Search for meaning			−0.07	0.05	−0.07
		Self-esteem			−0.43	0.06	−0.43^**^

Note: ^**^ *p* < 0.01

**Fig. 1:**
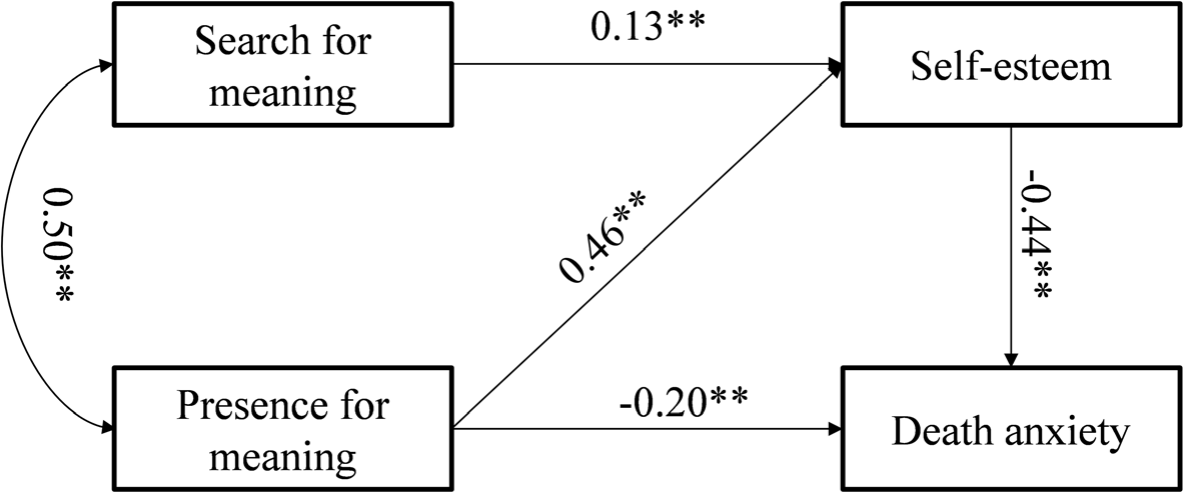
The final mediating effect model of self-esteem between meaning in life and death anxiety. Note: Search for meaning and Presence for meaning are two dimensions of meaning in life. All the coefficients are standardized, **p < 0.01

Next, path analysis was carried out in Amos 17.0. A partially mediated model containing mediators (self-esteem) and direct paths from search for meaning and presence of meaning to death anxiety was tested. The direct path from search for meaning and death anxiety was insignificant (β = − 0.07, *p* = 0.22) and thus deleted. The final model is presented in Fig. 1. This model exhibited a good fit with the data [χ^2^(*df* = 1) = 1.53, RMSEA = 0.02, SRMR = 0.046, CFI = 0.99], implying that self-esteem partially mediated the effect of presence of meaning on death anxiety and completely mediated the effect of search for meaning on death anxiety. Then, the mediating effects were tested for significance using the bootstrap estimation procedure in Amos 17.0 (a bootstrap sample of 1500 was specified). Table 3 shows the direct and indirect effects and their associated 95% confidence intervals. All confidence intervals did not include 0, indicating that all paths in the mediated model were significant. Taken together, these results reveal the mediating role of self-esteem in the relationship between meaning in life and death anxiety, supporting the hypothesis of the current study. According to literature requirements [40], no multicollinearity was apparent between independent variables; tolerance ranged between 0.62 and 0.72, and variance inflation factors ranged between 1.46 and 1.63.

**Table 3:** Direct and indirect effects of meaning in life on death anxiety

Model pathways	Estimated effect	Lower bonds	Up bonds
Direct effect			
Search for meaning →Self-esteem	.13	.06	.29
Presence for meaning → Self-esteem	.46	.27	.66
Presence for meaning → Death anxiety	−.20	−.34	−.11
Self-esteem→ Death anxiety	−.44	−.57	−.28
Indirect effect			
Search for meaning →Self-esteem→ Death anxiety	−.06	−.13	−.03
Presence for meaning →Self-esteem→ Death anxiety	−.20	−.35	−.10

## Discussion

This study aimed to identify a mediating role in the relationship between meaning in life and death anxiety in Chinese older adults. China has faced accelerated aging since the 1990s. The number of older adults (aged ≥65) increased from 62.99 million in 1990 to 140 million in 2015 (i.e., from 5.6% of the total population to 10.1%), indicating that China is now an aging society. There is no life without death; however, human beings possess the cognitive ability to be aware of their own mortality and to feel anxious regarding what may come afterwards. As individuals grow older, the specter of death begins to hover and prevents people from being completely free from death anxiety [22]. Furthermore, China is an atheistic country. According to national surveys conducted in the early twenty-first century, approximately 73.56% of China’s population (i.e., more than a billion people) are irreligious [41, 42]; the vast majority of Chinese participate in cosmological religion and exhibit no definitive religious beliefs, although religion may greatly relieve death anxiety [43]. Considering that China is home to one-fifth of the world’s older adults, many of whom possess no religious beliefs, it is highly important to study influential factors on death anxiety.

This study reveals that meaning in life is negatively correlated with death anxiety among older adults; higher scores on assessment dimensions regarding meaning in life reflected lower death anxiety. This finding is consistent with many prior studies. For instance, Ardelt also found meaning in life to be negatively associated with death anxiety among older adults [44]. Moore and colleagues determined that older adults with higher scores on meaning in life could face death optimistically even when suffering [45]. Older adults with higher meaning in life appear to clearly understand the meaning of life, interpret the essence of life based on their own values, and recognize the goals and values of their survival and life; therefore, they could look objectively at death, calmly accept death-related events, and experience less death anxiety [45]. As mentioned above, MMT suggests that the best way to reduce death anxiety is to facilitate acceptance of death and to seek the meaning of life. This theory suggests that a better understanding of the meaning and goals of life can reduce or eliminate anxiety and negative emotions related to death or near-death [24]. Results of this study demonstrate a negative correlation between meaning in life and death anxiety, which is consistent with MMT.

Our findings also revealed the mediating role of self-esteem between meaning in life and death anxiety. The two dimensions of meaning in life—presence of meaning and search for meaning—can each predict death anxiety through self-esteem, and self-esteem completely mediates the effect of search for meaning on death anxiety. These findings coincide with existing views. Scholars have suggested that meaning in life serves four important functions for humans, providing (1) life goals; (2) values and standards for the self-assessment of behavior; (3) a sense of control over life events; and (4) feelings of self-worth [46–49]. Self-esteem represents an overall assessment of the self and is therefore closely associated with these functions of meaning in life. People’s self-esteem can be enhanced through a sense of life value and the continuous pursuit of goals in life [34]. TMT, in seeking to minimize fear of death, assumes that cultural systems can serve as buffers against death anxiety. TMT holds that self-esteem is a feeling of “I am one valuable part of this meaningful world” [26, 50]. MMT, with the purpose of maximizing death acceptance and self-actualization, suggests that an individual can resist fear and anxiety surrounding death when he/she realizes the meaning of life and his/her personal worth [51]. Humans need self-esteem because the self-regulatory mechanism associated with it provides an elastic space that helps individuals alleviate anxiety [52]. According to these theories, when individuals ponder death-related issues, they contemplate their own worth (“Everyone eventually dies, so why are we alive, and what is the meaning of life?”) and become anxious about conflicting personal values. In this case, death anxiety can be relieved only if an individual realizes the values and meaning of life and feels that he/she is a valuable individual and is “one valuable part of this meaningful world,” which exemplifies self-esteem. That is, the experience and search for meaning in life by older adults should foster the realization that their lives are valuable and meaningful. This experience (i.e., self-esteem) further reduces anxiety caused by the reality that death is inevitable.

The findings of this study provide a more positive and hopeful perspective on death anxiety and carry important implications for practitioners working with people struggling with death anxiety. People may benefit from identifying or creating value and meaning in later stages of life, as evidenced by the current study; this notion coincides with the promotion of positive psychology and gradual reductions in death anxiety [53]. Thibault et al. suggested that practitioners should help patients seek or create value in later stages of life, however long that may be [54]. For example, people diagnosed with terminal cancer do not need to suffer from death anxiety and lose their remaining days waiting for death. Every person must face the looming threat of death; however, individuals can always unleash their inner potential to become a valuable part of a meaningful world (thus increasing their self-esteem) by constructing and seeking meaning for living. Such steps can transform death anxiety into a source of inspiration for an authentic life.

The current study also provides clinical implications related to counseling older adults who feel close to death. For example, staff who work in psychological counseling can help older adults to consolidate and enhance meaning in life by pointing out important life purposes and values, which will enable clients to enhance their self-esteem and serve as a buffer against death anxiety. In line with supporters of positive psychology, meaning-oriented interventions aimed at finding intentionality and goals may help individuals effectively reinforce coping strategies [55, 56]. Accordingly, psychological counselors could adopt interventions and counseling approaches to promote meaning in life and self-esteem to address death anxiety. Based on TMT, this type of clarity can be gained by assisting individuals in conforming to cultural and personal value standards and norms [23].

This study has some limitations. First, similar to related studies, a major limitation of this research is that findings relied on a cross-sectional sample of older adults; it was not a longitudinal study, which prevented the authors from establishing causal relationships regarding the concurrent effects of meaning in life and self-esteem on death anxiety. Future studies should test the presented mediating models using longitudinal approaches or a log linear method. Second, this study adopted a self-response method to measure death anxiety; responses may have been susceptible to social desirability effects (e.g., if respondents reported less death anxiety than they actually felt). Thus, more fully developed approaches can be used to replicate this method in subsequent studies and may reveal more interesting results. Third, we determined that self-esteem mediates the effect of meaning in life on death in older adults. Humans’ reactions to death anxiety are dynamic and multifaceted [4, 57, 58]; therefore, additional mediating factors may influence the relationship between meaning in life and death anxiety. Other active elements may thus serve protective functions against death anxiety, such as attribution styles and dispositional optimism, and should be considered in future studies. Last but not least, this study’s theoretical and empirical findings may oppose those of other studies, such as results suggesting that the direct effect of death salience may enhance death anxiety by eliciting regret and thoughts about the meaninglessness of death [31, 59]. This issue should be considered in future research.

## Conclusion

Death anxiety is a common phenomenon when older adults acknowledge their own mortality [60]. This study intended to explore potential solutions to relieve death anxiety in this population; findings indicated that self-esteem has a mediating effect in the connection between meaning in life and death anxiety in the Chinese elderly. These results provide additional insight into the limited body of work exploring the role of overall self-assessment in the connection between life goals and values and psychological distress that older adults experience as they realize that their lives are becoming increasingly short. Helping older adults consolidate and improve meaning in life should enable them to enhance their self-esteem and further alleviate death anxiety.

## Data Availability

Data is stored at the author’s university. The data is available from the corresponding authors on reasonable request.

## Abbreviations

MMT: Meaning Management Theory
TMT: Terror Management Theory
